# Chromosomal integration of Tn*5253* occurs downstream of a conserved 11-bp sequence of the *rbgA* gene in *Streptococcus pneumoniae* and in all the other known hosts of this integrative conjugative element (ICE)

**DOI:** 10.1186/s13100-021-00253-z

**Published:** 2021-11-05

**Authors:** Francesco Santoro, Valeria Fox, Alessandra Romeo, Elisa Lazzeri, Gianni Pozzi, Francesco Iannelli

**Affiliations:** Laboratory of Molecular Microbiology and Biotechnology (LAMMB), Department of Medical Biotechnologies, University of Siena, Siena, Italy

**Keywords:** Mobile genetic elements (MGE), Integrative conjugative element (ICE), Conjugative transposon, Conjugation, Attachment site, Circular form, Tn*5253*, *S. pneumoniae*

## Abstract

**Background:**

Tn*5253*, a composite Integrative Conjugative Element (ICE) of *Streptococcus pneumoniae* carrying *tet*(M) and *cat* resistance determinants, was found to (i) integrate at specific 83-bp integration site (*att*B), (ii) produce circular forms joined by a 84-bp sequence (*att*Tn), and (iii) restore the chromosomal integration site. The purpose of this study is to functionally characterize the *att*B in *S. pneumoniae* strains with different genetic backgrounds and in other bacterial species, and to investigate the presence of Tn*5253 att*B site into bacterial genomes.

**Results:**

Analysis of representative Tn*5253*-carryng transconjugants obtained in *S. pneumoniae* strains with different genetic backgrounds and in other bacterial species, namely *Streptococcus agalactiae*, *Streptococcus gordonii*, *Streptococcus pyogenes*, and *Enterococcus faecalis* showed that: (i) Tn*5253* integrates in *rbgA* of *S. pneumoniae* and in orthologous *rbgA* genes of other bacterial species, (ii) integration occurs always downstream of a 11-bp sequence conserved among streptococcal and enterococcal hosts, (iii) length of the *att*B site corresponds to length of the duplication after Tn*5253* integration, (iv) *att*B duplication restores *rbgA* CDS, (v) Tn*5253* produced circular forms containing the *att*Tn site at a concentration ranging between 2.0 × 10^−5^ to 1.2 × 10^−2^ copies per chromosome depending on bacterial species and strain, (vi) reconstitution of *att*B sites occurred at 3.7 × 10^−5^ to 1.7 × 10^−2^ copies per chromosome. A database search of complete microbial genomes using Tn*5253 att*B as a probe showed that (i) thirteen *att*B variants were present in the 85 complete pneumococcal genomes, (ii) in 75 pneumococcal genomes (88.3 %), the *att*B site was 83 or 84 nucleotides in length, while in 10 (11.7 %) it was 41 nucleotides, (iii) in other 19 bacterial species *att*B was located in orthologous *rbgA* genes and its size ranged between 17 and 84 nucleotides, (iv) the 11-bp sequence, which correspond to the last 11 nucleotides of *att*B sites, is conserved among the different bacterial species and can be considered the core of the Tn*5253* integration site.

**Conclusions:**

A functional characterization of the Tn*5253 att*B integration site combined with genome analysis contributed to elucidating the potential of Tn*5253* horizontal gene transfer among different bacterial species.

**Supplementary Information:**

The online version contains supplementary material available at 10.1186/s13100-021-00253-z.

## Introduction

The acquisition of new genetic material by horizontal gene transfer (HGT) significantly drives bacterial genome evolution and is mediated by Mobile Genetic Elements (MGEs). The term “mobilome” is used to indicate the entire set of MGEs of the microbiome[1]. MGEs are responsible for the spread of resistance and virulence genes in the microbial communities [2–4]. Thus, to study the acquisition and dissemination of antibiotic determinants in a bacterial population, the characterization of mobilome is crucial [5]. Even though new metagenomic approaches, both whole and targeted [1, 6, 7] have been implemented, a functional study of MGEs is still required [8, 9]. Integrative and Conjugative Elements (ICEs) are MGEs commonly found in bacteria where they can constitute up to 25 % of the genome [5, 10–14]. One of the most studied ICE of gram-positive bacteria is Tn*916*, a conjugative transposon originally found in *Enterococcus faecalis* which carries the *tet*(M) tetracycline resistance gene and is considered the prototype of the Tn*916*-Tn*1545* family of ICEs [15–19]. Conjugative transposons of the Tn*916*-Tn*1545* family can insert at multiple integration sites in the chromosome [20], while other ICEs, like Tn*5253*, SXT, Tn*5397*, and ICE*St1*, integrate at a single specific site [21–26]. We previously characterized Tn*5253*, a 64,528-bp composite ICE of *Streptococcus pneumoniae*, containing the ICE Tn*5251* of the Tn*916*-Tn*1545* family and the Ω*cat*(pC194) element carrying *tet*(M) and *cat* resistance determinants, respectively [27–29]. Tn*5253* was found integrated at 83-bp specific integration site (*att*B) located in the essential gene *rbgA* of the *S. pneumoniae* chromosome [26, 28, 30]. The ICE was shown to excise from the pneumococcal chromosome with production of (i) circular forms in which the ends of the element were joined by a 84-bp sequence (*att*Tn) and (ii) a reconstituted chromosomal *att*B. Tn*5253*, once integrated into the chromosome, was flanked by the *att*L site, identical to *att*B, and the *att*R site, identical to *att*Tn. Pneumococcal mobilome analysis showed the frequent presence of Tn*5253*-like elements in multidrug-resistant *S. pneumoniae* strains and the maintenance of the element in all derivative isolates [31–34]. In this work, in order to contribute to mobilome characterization, we first conducted a functional characterization of the Tn*5253* integration site, by analyzing *att*B in Tn*5253*-carrying transconjugants obtained in *S. pneumoniae* strains with different genetic backgrounds and in strains belonging to other bacterial species. We then investigated the presence of the Tn*5253 att*B site into the complete microbial genomes available in public databases.

## Results and discussion

### Tn*5253* integration sites and circularization in different pneumococcal transconjugants

**Fig. 1:**
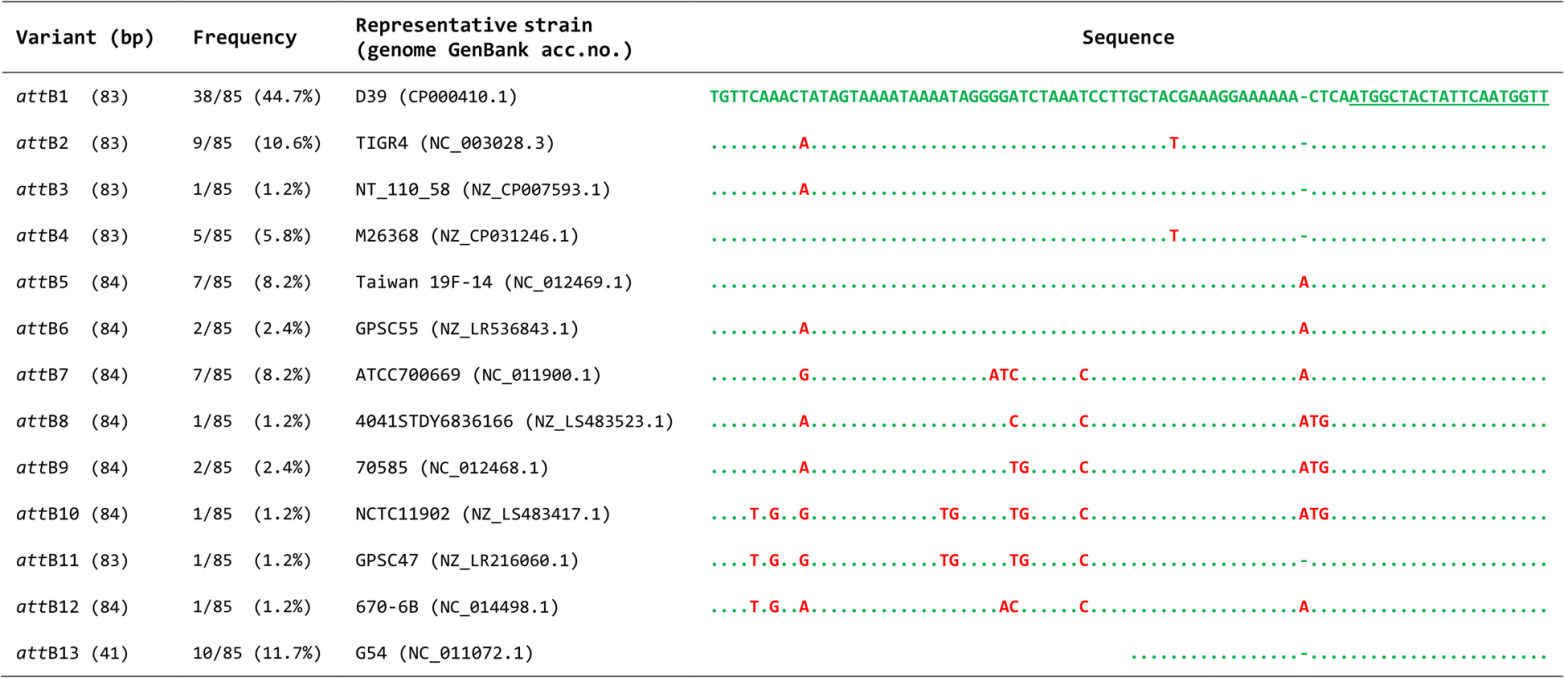
Allelic variants of the Tn5253 *att*B integration site in *S. pneumoniae*. Tn5253 *att*B is located in the essential pneumococcal *rbg*A gene. In the 85 complete *S. pneumoniae* genomes, 13 allelic variants of *att*B were found. The 83-bp variant 1 (*att*B1) is the most frequent, carried by 38 genome strains (44.7 % of the analyzed genomes), including D39 strain, its rough derivative R6, and A66 strain, and was used as reference for the alignment. Variant 2 is carried by TIGR4 and other 8 genome strain and contains 2 nucleotide substitutions. BM6001, the original Tn*5253*-carrying clinical strain, and DP1322, in which Tn*5253* was transferred by transformation from BM6001, harbour variant 5. SP18-BS74, whose draft genome is available, harbours the 41-bp variant 13. Within the sequences, identical nucleotides are indicated by periods, substitutions in red. For better alignment, dashes are inserted. The 20 nucleotides belonging to the *rbg*A coding sequence are underlined

Representative Tn*5253*-carrying transconjugants were obtained in *S. pneumoniae* with different genetic backgrounds, namely TIGR4, A66 and SP18-BS74 [28] (Table 1). DNA sequence analysis of Tn*5253*-chromosome junction fragments showed that: (i) Tn*5253* integration occurred at a specific integration site (*att*B) located in *rbgA* gene of the pneumococcal chromosome [26], (ii) *att*L was identical to *att*B and (iii) *att*R was identical to *att*Tn, as already described for D39 and its derivative strains [26], and that (iv) *att*B sites among these pneumococcal strains were not identical, with their size varying from 41 nucleotides (variant *att*B13 in SP18-BS74) to 83 nucleotides (variant *att*B2, in TIGR4 and A66) (Fig. 1). We also analysed the nucleotide sequence of Tn*5253* junction fragments in the original Tn*5253*-carrying clinical strain BM6001 and DP1322, in which Tn*5253* was transferred by transformation of a crude lysate from BM6001 [35]. *att*L sequences of BM6001 and DP1322 were identical and belonged to a 84 bp-long variant (*att*B5, Fig. 1), since Tn*5253* integration occurred via homologous recombination between DNA sequences beyond Tn*5253 att* sites. Tn*5253* was found to excise from pneumococcal chromosome with consequent production of circular forms, containing the *att*Tn site, and reconstitution of *att*B site [26]. To investigate if different pneumococcal genetic backgrounds influence the excision and circularization of Tn*5253*, quantitative PCR on cell lysates was used to quantify the excision of Tn*5253* and *att*B reconstitution in liquid pneumococcal cultures (Table 2). Interestingly, the transconjugant FR56, derived from SP18-BS74, produced Tn*5253* circular forms and reconstituted *att*B site at very high frequency (1.2 × 10^−2^ and 1.9 × 10^−3^ copies per chromosome, respectively). However, these results did not correlate with the conjugation frequency, which was 6.1 × 10^−6^, indicating that the frequency of circularization is not the only limiting factor of the conjugation process. Neither circular forms nor reconstituted *att*B of Tn*5253* could be detected in the TIGR4 background (<3.6 × 10^−5^ and to <3.5 × 10^−4^, respectively), correlating with the absence of conjugal transfer (<9.9 × 10^−8^). Analysis of Tn*5253* integration, in pneumococci with different genetic backgrounds, revealed that the element always integrates downstream of nucleotide position 20 of *rbgA* coding sequence (CDS) (Fig. 1). *rbgA* is an essential gene encoding the ribosomal biogenesis GTPase protein involved in the 50S ribosome subunit assembly [36]. Integration of Tn*5253* leads to the duplication of the integration site restoring the CDS and preserving cell viability. Site specific integration of MGEs often occurs at the 5’ or 3’ end of genes, such as those coding for tRNAs or ribosomal proteins, which are essential and conserved among different bacterial species. This characteristic allows to overpass the single species border and favors the spread of MGEs within bacterial communities.

**Table 1:** Bacterial strains and relevant properties

Strain	Relevant properties^a^	Genome Genbank acc. no., [Reference]
	*Streptococcus pneumoniae*	
A66	Avery’s strain, clinical isolate, serotype 3	LN847353.1, draft genome, [41, 42]
HB565	A66 derivative, carrying *str-1*, Sm^R^	[39, 43]
FR39	HB565 transconjugant derivative, carrying Tn*5253*, Sm^R^, Tc^R^, Cm^R^	This study
TIGR4	Clinical isolate, serotype 4	NC_003028.3, [44]
FP47	TIGR4 derivative, carrying *nov-1*, Nov^R^	[29]
FR54	FP47 transconjugant derivative, carrying Tn*5253*, Nov^R^, Tc^R^, Cm^R^	[29]
SP18-BS74	Clinical isolate, serotype 18 C	NZ_ABAE01000001.1, draft genome, [45]
FR55	SP18-BS74 derivative, carrying *str-1*, Sm^R^	[28]
FR56	FR55 transconjugant derivative, carrying Tn*5253*, Sm^R^, Tc^R^, Cm^R^	[28]
	Other streptococci	
H36B	*S. agalactiae*, clinical isolate, serotype Ib	NZ_LN847353.1, [46]
FR67	H36B transconjugant derivative, carrying Tn*5253*, Tc^R^, Cm^R^	[29]
SF370	*S. pyogenes*, clinical isolate, serotype M1	AE004092.2, [47]
FR68	H36B transconjugant derivative, carrying Tn*5253*, Tc^R^, Cm^R^	[29]
V288	*S. gordonii* Challis, clinical isolate	NC_009785.1, [48, 49]
GP204	V288 derivative, carrying *str-204*; Sm^R^	[50]
FR43	GP204 transconjugant derivative, carrying Tn*5253*, Sm^R^, Tc^R^, Cm^R^	[29]
	*Enterococcus faecalis*	
OG1	Clinical isolate, formely named 2SaR	[51]
OG1RF	OG1 derivative, Fus^R^, Rif^R^	NC_017316.1, [52, 53]
OG1SS	OG1 derivative, Spe^R^, Sm^R^	[15, 54]
FR49	OG1SS transconjugant derivative, carrying Tn*5253*, Spe^R^, Sm^R^, Tc^R^, Cm^R^	[28]
JH2	Clinical isolate	[55]
JH2-2	JH2 derivative, Fus^R^, Rif^R^	NZ_KI518257.1, draft genome, [55]
FR50	JH2-2 transconjugant derivative, carrying Tn*5253*, Fus^R^, Rif^R^, Tc^R^, Cm^R^	[28]

^a^ *str-41* and *str-204* indicate point mutations conferring resistance to streptomycin, while *nov-1* to novobiocin. Sm, streptomycin; Tc, tetracycline,

Cm, chloramphenicol; Nov, novobiocin; Fus, fusidic acid; Rif, rifampicin; Spe, spectinomycin;

**Table 2:** PCR quantification of Tn*5253* circular forms and reconstituted *att*B integration sites in Tn*5253*-carrying transconjugants

Strain	Genetic background	Circular Forms	Reconstituted *att*B Site	Conjugation Frequency Mean (Range)^a^
FR39	*S. pneumoniae* A66	2.0 × 10^−5^ ± 1.9 × 10^−5^	<6.9 × 10^−6^ ± 7.1 × 10^−8^	4.4 × 10^−7^ (3.2 × 10^−7^ - 5.8 × 10^−7^)
FR54	*S. pneumoniae* TIGR4	<3.6 × 10^−5^ ± 2.6 × 10^−6^	<3.5 × 10^−4^ ± 2.6 × 10^−5^	<9.9 × 10^−8^ (<9.6 × 10^−8^ - <1.3 × 10^−7^)
FR56	*S. pneumoniae* SP18-BS74	1.2 × 10^−2^ ± 7.5 × 10^−5^	1.9 × 10^−3^ ± 1.0 × 10^−4^	6.1 × 10^−6^ (3.5 × 10^−6^ - 9.5 × 10^−6^)
FR67	*S. agalactiae* H36B	2.9 × 10^−5^ ± 8.7 × 10^−6^	3.0 × 10^−4^ ± 1.0 × 10^−4^	1.1 × 10^−6^ (3.2 × 10^−7^ - 2.1 × 10^−6^)
FR68	*S. pyogenes* SF370	3.0 × 10^−5^ ± 1.0 × 10^−5^	3.7 × 10^−5^ ± 6.2 × 10^−6^	9.5 × 10^−4^ (3.3 × 10^−5^ - 2.6 × 10^−3^)
FR43	*S. gordonii* V288	6.5 × 10^−5^ ± 4.4 × 10^−5^	7.9 × 10^−5^ ± 1.2 × 10^−5^	8.3 × 10^−7^ (1.2 × 10^−7^ - 2.0 × 10^−6^)
FR49	*E. faecalis* OG1SS	1.4 × 10^−4^ ± 9.2 × 10^−5^	6.8 × 10^−3^ ± 1.0 × 10^−4^	<1.8 × 10^−8^ (<1.0 × 10^−9^ - <3.9 × 10^−8^)
FR50	*E. faecalis* JH2-2	<2.7 × 10^−7^ ± 7.5 × 10^−8^	1.7 × 10^−2^ ± 1.3 × 10^−3^	<2.7 × 10^−8^ (<9.9 × 10^−9^ - <5.0 × 10^−8^)

^a^Frequency refers to mating experiments where *S. pneumoniae* FP10 or FP11was the conjugation recipient [29]; conjugation frequency is expressed as

CFU of transconjugants per CFU of donors; each result is the mean of at least three mating experiments

### Tn*5253* integration sites and circularization in *Streptococcus* and *Enterococcus*.

**Fig. 2:**
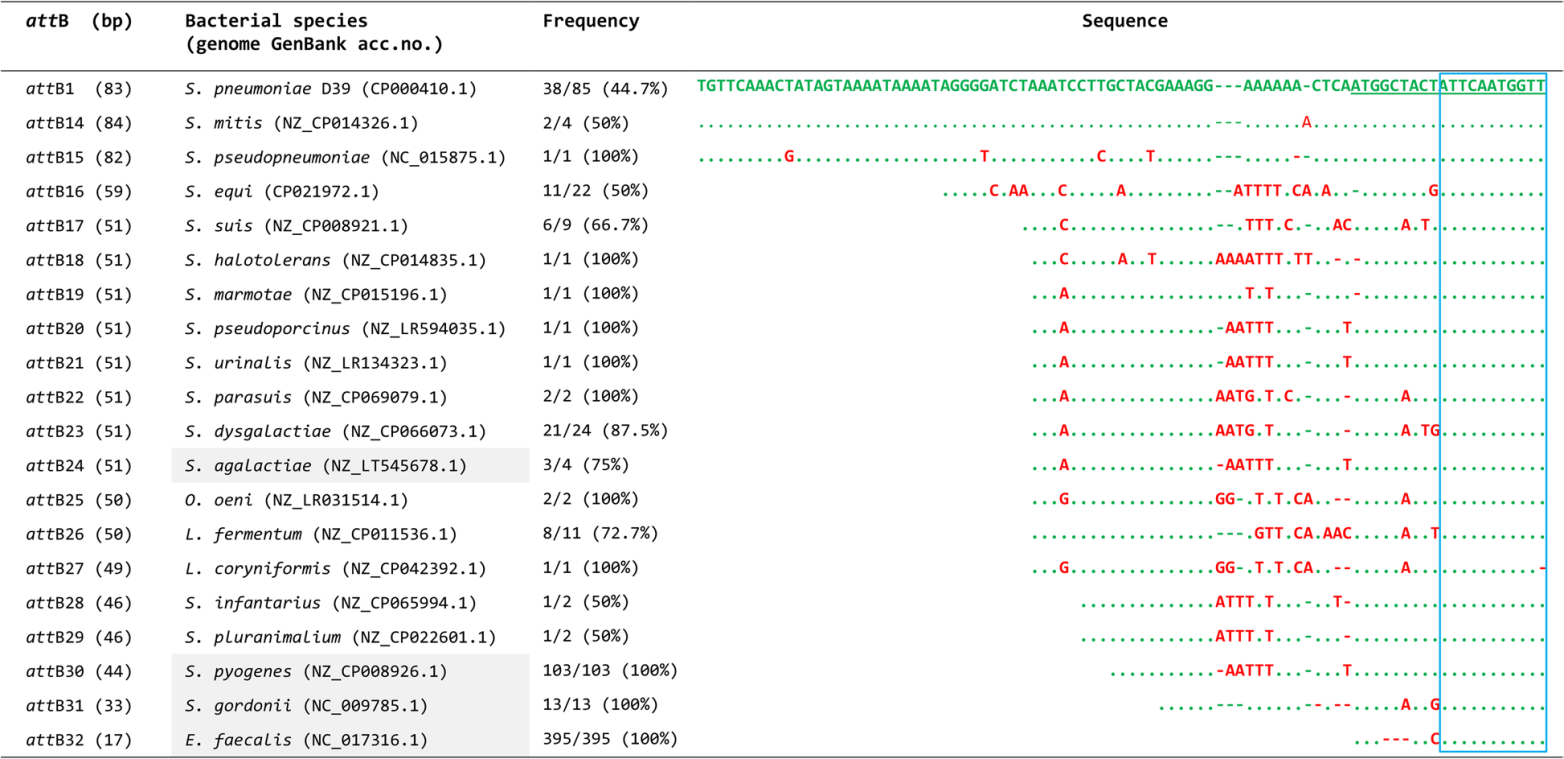
Tn*5253 att*B integration sites in the orthologous *rbg*A genes of other bacterial species. Genome sequence analysis identified Tn*5253 att*B sites in the orthologous *rbg*A genes of 18 other bacterial species with a size ranging between 33 nucleotides of *S. gordonii* to 84 nucleotides of *Streptococcus mitis*. The 17-bp *E. faecalis att*B was at first experimentally found by PCR and sequencing the Tn*5253*-chromosomal junction fragments of our *E. faecalis* transconjugants. Then the 17 nucleotides were used as probes for database interrogation. Inside the same bacterial species, different strains can harbour different allelic variants (up to 7 in *S. equi*). The sequence of the most represented allelic variant was used for the sequence alignment and its frequency is reported. The *S. pneumoniae* D39 *att*B variant 1 was used as reference. Tn*5253* chromosomal integration, in the original *S. pneumoniae* host, as in the other functionally characterized streptococcal and enterococcal hosts (shaded), occurs always downstream of a 11-bp conserved sequence, namely the last 11 nucleotides of *att*B sites. These 11 nucleotides (boxed in blue) are conserved also among the *att*B sites of other bacterial species. Within the sequences, identical nucleotides are indicated by periods, substitutions are in red. For better alignment, dashes are inserted. The 20 nucleotides belonging to the *rbg*A coding sequence are underlined

**Fig. 3:**
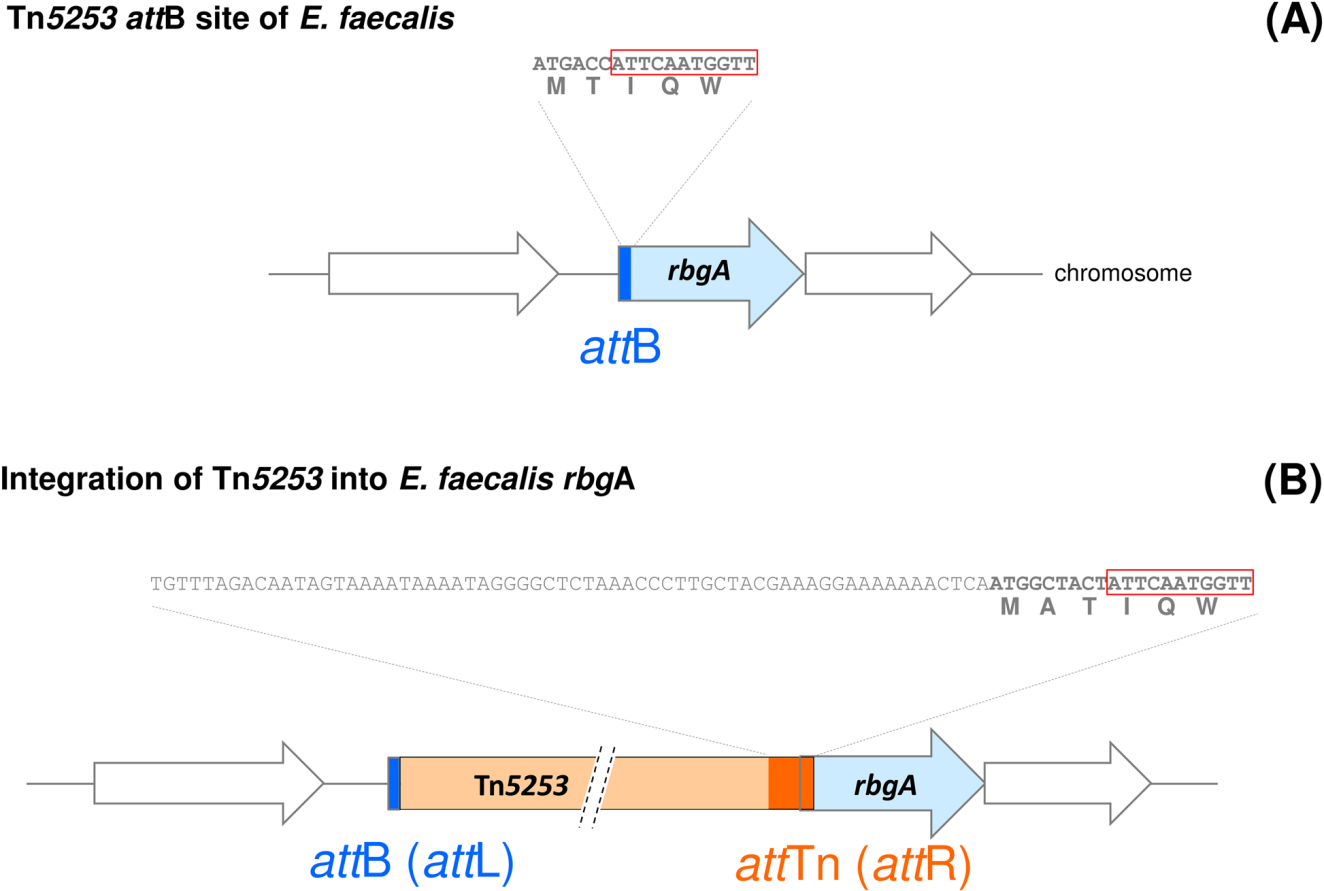
Tn*5253 att*B site and integration in *E. faecalis* chromosome. (A) Tn*5253 att*B (represented as a blue box) in *E. faecalis* is composed by 17 nucleotides which correspond to the first 17 nucleotides of the *rbg*A CDS (light blue arrow). In streptococci and enterococci, Tn*5253* always integrates downstream of a conserved 11-nucleotides sequence (boxed in red). The nucleotide sequence of *att*B and deduced amino acid sequence are reported. (B) Site specific integration of Tn*5253* into *rbg*A causes integration site duplication, restoring an intact CDS. The integration of Tn*5253* into bacterial chromosome seems to be polarized, since *att*Tn (orange box) always flanks the element (light orange box) at the right end. In *E. faecalis* integration site duplication results in the acquisition of an additional codon (GCT ◊ Alanine) in the *rbg*A CDS. *att*B and *att*Tn sites are not scaled. The 84 nucleotides sequence of *att*Tn and the deduced amino acid sequence of RbgA N-terminal end are reported. Amino acids single-letter code is used

We then extended Tn*5253* functional analysis to streptococci and enterococci characterizing Tn*5253* circular forms and integration sites in the transconjugants obtained in *S. agalactiae* H36B, *S. pyogenes* SF370, *S. gordonii* V288, *E. faecalis* OG1SS and JH2-2 backgrounds (Table 1). In all bacterial hosts, Tn*5253* integration occurred in the orthologous *rbg*A genes (Fig. 2). As found in *S. pneumoniae*, in all the bacterial hosts tested: (i) *att*L was identical to *att*B regardless of the bacterial strain harbouring the element and *att*R was identical to *att*Tn suggesting a polarization of Tn*5253* integration process, (ii) integration always occurs downstream of a 11-bp conserved sequence, namely the last 11 nucleotides of *att*B sites, (iii) length of the *att*B site corresponds to length of the duplication after Tn*5253* integration, (iv) *att*B site duplication restores *rbgA* CDS. It is worth to note that in *E. faecalis*, *att*B duplication modifies the *rbgA* predicted gene product (Fig. 3). Tn*5253* produced circular forms at a similar frequency in *S. agalactiae* (2.9 × 10^−5^ copies per chromosome) and *S. pyogenes* (3.0 × 10^−5^ copies per chromosome), while no circular forms were detected in *E. faecalis* JH2-2 genetic background (<2.7 × 10^−7^ copies per chromosome) (Table 2). Reconstituted *att*B sites were found in all streptococci tested at a frequency ranging between 3.7 × 10^−5^ (in *S. pyogenes*) to 1.7 × 10^−2^ (in *E. faecalis* JH2-2 background) copies per chromosome. In *E. faecalis*, Tn*5253* excision and circularization are strain dependent: a representative transconjugant obtained in OG1SS background produced circular forms and reconstituted *att*B site at 1.4 × 10^−4^ and 6.8 × 10^−3^ copies per chromosome, respectively; transconjugant FR50, obtained in JH2-2 background, produced reconstituted *att*B site at a frequency of 1.7 × 10^−2^ copies per chromosome but did not produce circular forms (<2.7 × 10^−7^). Conjugation frequency was lower than circularization frequency in all the tested strains except in *S. pyogenes* FR68. Many other factors are likely to be important in the conjugation process such as the expression of a capsular polysaccharide [37], the cell wall thickness, the surface charges, and the ability of the conjugation pore to establish a stable contact between cells from different species.

### Genome sequence analysis of Tn*5253 att*B site in *S. pneumoniae*

To integrate biological data, a genome-wide investigation of Tn*5253 att*B among pneumococci was carried out. The database of 85 complete *S. pneumoniae* genomes (accessed in August 2021) was interrogated by using as a query the 83-bp *att*B. Sequence homology analysis identified thirteen allelic variants of *att*B. (Fig. 1, Table S1). In 75 genomes (88.3 %), the *att*B site was 83 or 84 nucleotides in length, while in 10 (11.7 %) it was 41 nucleotides. The 83-bp *att*B variant 1 is the most frequent variant, carried by 38 genome strains (44.7 % of the analyzed genomes), including D39 strain, its rough derivative R6, and the classical type 3 Avery’s strain A66. Variant 2 is carried by TIGR4 and other 8 genome strains (10.6 %) and contains two nucleotide substitutions. Variant 13 is harboured by G54 and other 9 genome strains (11.7 %) and contains only the last 41 nucleotides of variant 1. In addition, SP18-BS74, whose draft genome is available, also harbours variant 13. Variants 5 and 7 are carried by 7 strains (8.2 %), variant 4 by 5 strains (5.8 %), variants 6 and 9 by 2 strains (2.4 %). The remaining 5 variants (3, 8, 10, 11 and 12) were found in only in one strain. In thirteen pneumococcal genomes, carrying the *att*B variants 1, 2, 7, 11 and 12, Tn*525*3-like elements were integrated into the pneumococcal chromosome, resulting in the duplication of the *att*B site.

### Genome sequence analysis of Tn*5253 att*B site in other bacterial species

Genome analysis was extended to the 58,138 complete microbial genomes (accessed in August 2021). Homology search identified the Tn*5253 att*B site in 18 other bacterial species, including the functionally characterized *S. agalactiae*, *S. pyogenes*,*S. gordonii*, hosts (Fig. 2, Table S2). The 17-bp *E. faecalis att*B was at first experimentally found by sequencing the Tn*5253*-chromosomal junction fragments obtained by inverse PCR from our *E. faecalis* transconjugants. Then the 17 nucleotides were used as a probe for database interrogation. Tn*5253 att*B was located in orthologous *rbgA* genes and its size ranged between 17 nucleotides of *E. faecalis* to 84 nucleotides of *Streptococcus mitis*. Alignment of the *att*B sequences obtained from the different bacterial species confirms the presence of the 11-bp conserved sequence. Theoretically, all of these *att*B sites allow Tn*5253* integration, however only in one genome, namely *Streptococcus mitis* SVGS_061, a Tn*525*3-like element was found integrated, producing *att*B duplication.

## Conclusions

In the present paper we conducted a functional characterization of Tn*5253 att*B site in *S. pneumoniae* and other streptococcal and enterococcal species and found that: (i) during conjugal transfer, Tn*5253* integrated in *S. pneumoniae rbgA* gene or in the orthologous *rbgA* genes of the other bacterial hosts, (ii) Tn*5253* produced circular forms containing the *att*Tn site and the frequency was species- and strain-dependent, (iii) reconstitution of *att*B site was species- and strain-dependent. Through a DNA homology search conducted in the complete microbial genome database, we also found that: (i) thirteen allelic variants of the Tn*5253 att*B site were present in the complete *S. pneumoniae* genomes and their size ranged from 41 to 84 nucleotides, (ii) in other bacterial species, Tn*5253 att*B is located in orthologous *rbgA* genes with a size ranging between 17 and 84 nucleotides. Tn*5253* integration, in the original *S. pneumoniae* host, as in the other streptococcal and enterococcal hosts, occurs always downstream of a 11-bp conserved sequence located in the *rbg*A CDS. Genome analysis revealed that the 11 nucleotides, corresponding to the last 11 nucleotides of the *att*B sites, are conserved also among the *att*B sites of other bacteria and can be considered the core of the integration site. In conclusion, even if a huge number of bacterial genomes is available, an *in-silico* analysis and a functional characterization of the mobilome is reported only in few cases. In this work, a functional characterization of the Tn*5253 att*B integration site, combined with genome sequence analysis, contributed to elucidating the potential of Tn*5253* horizontal gene transfer among different bacterial species.

## Materials and methods

### Bacterial strains, growth, and mating conditions

Bacterial strains and their relevant properties are reported in Table 1. Both streptococcal and enterococcal strains were grown in tryptic soy broth or tryptic soy agar (Difco) supplemented, where appropriate, with antibiotics. Plate mating conjugation experiments were performed as previously described [38]. Briefly, donor and recipient cells were grown until the end of exponential phase and mixed at a 1:10 ratio, then were collected by centrifugation, plated and incubated for 4 h. Cells were harvested by scraping the plates and recombinant strains were selected by a multilayer plating procedure in presence of the appropriate antibiotics. Transconjugant FR39 was obtained from a mating experiment where FP58 [29] was the donor of Tn*5253* and HB565, a streptomycin resistance derivative of type 3 Avery strain A66 [14, 39, 40], was the recipient.

### Bacterial lysate preparation

Bacterial cultures (1 ml) were harvested in exponential phase (OD_590_ about 0.2, roughly corresponding to 5 × 10^8^ CFU/ml) and centrifuged at 11,000 x *g* for 2 min. Pneumococcal lysates were obtained by using lysis solution (0.1 % DOC, 0.008 % SDS) as already reported [26]. Streptococcal and enterococcal cell pellets were resuspended in 90 µl protoplasting buffer (25 % sucrose, 100 mM Tris pH 7.2, 5 mM EDTA), then lysozyme (for *E. faecalis*) or mutanolysin (for *S. agalactiae*, *S. gordonii* and *S. pyogenes*) was added at a final concentration of 1 mg/ml or 20 µg/ml respectively and mixtures were incubated at 37 °C for 1 h. Protoplasts were centrifuged at 3,000 x *g* for 15 min, resuspended in 100 µl of dH_2_O, heated at 85 °C for 5 min and kept on ice until use.

### PCR, inverse PCR, sequencing

PCR experiments and direct DNA sequencing of PCR amplicons were carried out essentially as already described [28, 29]. Briefly, PCR reactions were carried out in a 25-µl reaction mixture containing DreamTaq buffer 1X, 100 µM dNTPs, 1.5 mM MgCl_2_, 10 pmol of each primer, 0.2 U of DreamTaq enzyme, 1 µl bacterial culture. Inverse PCR, for amplifying the Tn*5253*-chromosome junctions, was performed with pairs of divergent primers targeting the Tn*5253* ends as described [29]. 100 ng of each unpurified PCR fragment were used as template in sequencing reactions carried out with the BigDye Terminator v3.1 Cycle Sequencing Kit.

### Quantitative Real time PCR

A LightCycler 1.5 apparatus (Roche) and the KAPA SYBR FAST qPCR kit Master Mix Universal (2X) (Kapa Biosystems) were used for Real Time PCR experiments according to the protocol extensively described [26]. Quantification of Tn*5253* circular intermediates and reconstituted pneumococcal *att*B was obtained with the primer pairs IF327/IF328 and IF496/IF356, respectively [26]. Reconstituted *att*B site was quantified in *S. agalactiae* with the primer pair IF560/IF561 which amplified a 353 bp fragment, in *S. gordonii* with IF544/IF545 which amplified a 396 bp fragment, in *S. pyogenes* with IF509/IF510 which amplified a 249 bp fragment, in *E. faecalis* with IF525/IF532 which amplified a 480 bp fragment (Table S3). A standard curve for the *gyrB* gene was used to standardize results and melting curve analysis was performed to differentiate the amplified products from primer dimers as reported [26].

### Microbial database interrogation and sequence analysis

Homology searches of the databases available at the National Center for Biotechnology Information were conducted using the Microbial Nucleotide BLAST (https://blast.ncbi.nlm.nih.gov/Blast.cgi?PAGE_TYPE=BlastSearch&BLAST_SPEC.

=MicrobialGenomes), selecting the complete genomes database. Default parameters were used and only alignments with significant e-values were considered. We built a stand-alone database containing only genomes of interest to be searched with BLAST software to confirm the results. Sequence analysis was carried out with BioEdit 7.2.5 (http://bioedit.software.informer.

com/). Multiple DNA sequence alignments were performed using Clustal Omega (https://www.ebi.ac.uk/Tools/msa/clustalo/).

## Supplementary Information

**Additional file 1: Table S1.** Blast searches output of the *S. pneumoniae* complete genomes. For each variant, name, length, sequence, strain host, GenBank accession number, are reported.

**Additional file 2: Table S2.** Blast searches output of the complete microbial genomes. For each *att*B site, name, length, sequence, bacterial species host, GenBank accession number, are reported.

**Additional file 3: Table S3.** Oligonucleotide primers.

**Additional file 4: Table S4.** Quantitative PCR data. The threshold cycles (Cts) relative to circular forms (CI), reconstitution of *att*B site, and chromosomal reference gene (*gyrB*) quantification are reported. Standard curves, slope and intercept values are reported.

## Data Availability

All data generated or analyzed during this study are included in this published article.
