# Supplementary material for: Chromosomal integration of Tn5253 occurs downstream of a conserved 11-bp sequence of the rbgA gene in Streptococcus pneumoniae and in all the other known hosts of this integrative conjugative element (ICE)
Source: Mob DNA. 2021 Nov 5;12:25. doi: 10.1186/s13100-021-00253-z (PMC8571831; doi:10.1186/s13100-021-00253-z)
Supplement: Supplementary file 3 — Additional file 3: Table S3. Oligonucleotide primers. [file 13100_2021_253_MOESM3_ESM.docx]

**Table S3.** Oligonucleotide primers.

| Name | Sequence (5’to 3’) | GenBank ID: nucleotides |
| --- | --- | --- |
| *Streptococcus agalactiae* | | |
| IF560 | Aac gaa acc tat cag cgg aa | AAJS01000088: 6,047-6,028 |
| IF561 | TTT GGG TTT GTC TCC GAC GA | AAJS01000088: 5,695-5,714 |
| IF927 | ACA AGC GAG AAG GTC AAG AAG TT | AAJS01000029: 4,775-4,797 |
| IF928 | GTG TCA AGG CAG TAC GAA ATC | AAJS01000029: 5,012-5,032 |
| *Streptococcus gordonii* | | |
| IF512 | TGC TTT AGG AGA TGT TGA GTT | CP000725: 1,253,207-1,253,187 |
| IF513 | ACC GCA GAC TGT TCT TTA GA | CP000725: 1,252,812-1,252,831 |
| IF544 | CAG ATG GAG AAA TGG AAG AT | CP000725: 1,483,666- 1,483,647 |
| IF545 | GCT GTA CGG AAA CCT TGC TC | CP000725: 1,483,531-1,483,550 |
| *Streptococcus pyogenes* | | |
| IF509 | AAG TAG AAA TGG CGA AGT GAA | AE004092: 953,960-953,980 |
| IF510 | GAC TAG AAA GTG GTA AGC GT | AE004092: 954,208-954,189 |
| IF306 | AAG GTT TGA CGG CGG TAA | AE004092: 582,657-582,674 |
| IF307 | ACG AGC AAC TTG TGG GTT | AE004092: 582,814-582,797 |
| *Enterococcus faecalis* | | |
| IF525 | GGT TAC GGG AAG AAA GCG GT | CP002621: 1,430,659-1,430,678 |
| IF532 | GCC TAT GGG ATT GCT ACA CC | CP002621: 1,431,138-1,431,119 |
| IF943 | ACC AAG AAT ATC GTC GTG GT | CP002621: 5,120-5,139 |
| IF944 | AAT TGA AAT GTG TAA GCC TCG | CP002621: 5,310-5,290 |
